# Resolvin D1 Alleviates Ventilator-Induced Lung Injury in Mice by Activating PPAR*γ*/NF-*κ*B Signaling Pathway

**DOI:** 10.1155/2019/6254587

**Published:** 2019-06-04

**Authors:** Haifa Xia, Jingxu Wang, Shujun Sun, Fuquan Wang, Yiyi Yang, Lin Chen, Zhipeng Sun, Shanglong Yao

**Affiliations:** ^1^Institute of Anesthesia and Critical Care Medicine, Union Hospital, Tongji Medical College, Huazhong University of Science and Technology, Wuhan 430022, China; ^2^Department of Anesthesia, Union Hospital, Tongji Medical College, Huazhong University of Science and Technology, Wuhan 430022, China; ^3^Department of Anesthesia, Wuhan Children's Hospital (Wuhan Maternal and Child Healthcare Hospital), Tongji Medical College, Huazhong University of Science and Technology, Wuhan 430016, China

## Abstract

As one of the basic treatment modalities in the intensive care unit (ICU), mechanical ventilation can cause or aggravate acute lung injury or ventilator-induced lung injury (VILI). Resolvin D1 (RvD1) is an endogenous polyunsaturated fatty acid derivative with strong anti-inflammatory action. In this study, we explored if RvD1 possesses a protective effect on VILI. Mice were ventilated with high tidal volume (40 mL/kg, HV_T_) for 4 h and were then intraperitoneally administered RvD1 at the beginning of high tidal volume ventilation and given GW9662 (a PPAR-*γ* antagonist) intraperitoneally 30 min before ventilation. RvD1 attenuated VILI, as evidenced by improved oxygenation and reduced histological injury, compared with HV_T_ -induced lung injury. Similarly, it could ameliorate neutrophil accumulation and production of proinflammatory cytokines in lung tissue. In contrast, the protective effect of RvD1 on lung tissue could be reversed by GW9662. RvD1 mitigated VILI by activating peroxisome proliferator-activated receptor gamma (PPAR-*γ*) and inhibiting nuclear factor-kappa B (NF-*κ*B) signaling pathways in mice. In conclusion, RvD1 could reduce the inflammatory response in VILI by activating PPAR-*γ* and inhibiting NF-*κ*B signaling pathways.

## 1. Introduction

Mechanical ventilation (MV), an essential technology in the intensive care unit (ICU), as a life-saving measure, is extensively applied in patients with pulmonary dysfunction or injury. MV can cause and aggravate lung injury, thereby putting patients at risk of ventilator-induced lung injury (VILI), which can lead to increased morbidity and mortality [1–3], especially when the therapeutic focus is mainly directed at inflammation inhibition, since VILI is characterized by destruction of the alveolar-capillary barrier and increased permeability, resulting in edema, inflammatory leukocyte infiltration (predominantly neutrophils), and bleeding [3, 4]. Specialized proresolving lipid mediators, including the lipoxins, resolvins, protectins, and maresins, were identified by Serhan et al. and they have offered a novel way to resolve inflammation [5–10]. Resolvin D1 (RvD1) was stereochemically assigned as 7S, 8R, 17S-trihydroxy-4Z, 9E, 11E, 13Z, 15E, and 19Z-docosahexaenoic acid [11], and previous studies showed that it possessed effective and stereoselective anti-inflammatory effects, such as limiting neutrophil infiltration and promoting apoptosis [12]. Several studies indicated that RvD1 worked on a number of pulmonary inflammatory conditions, including cystic fibrosis lung disease [13], asthma [14], and ALI (induced by LPS) [15]. RvD1 promotes inflammatory resolving mainly by reducing neutrophil infiltration, inhibiting the interaction between neutrophils and platelets, and enhancing restitution of barrier integrity [15]. In addition, it was found that RvD1 could directly act on the single cell level to prevent neutrophils migration to the endogenous chemokine interleukin-1*β*as well as enhance the phagocytosis of human macrophages [16, 17]. Meanwhile, RvD1 reduces endothelium-dependent nitric oxide production, and expression of leukocyte adhesion receptors directly regulates endothelial interactions in vivo. As we all know, aggregation of neutrophils, disruption of alveolar-capillary barrier, and release of proinflammatory factors inflammatory cytokines are all characteristics of inflammation induced by VILI [18]. Therefore, the present study was undertaken to investigate the role of RvD1 associated mechanism in the resolution of VILI. What is more, it has been proved that lipoxins, a kind of proresolving lipid mediators as well as RvD1, exert their anti-inflammatory effects in VILI [19]. Therefore, the question as to whether RvD1 could exert protective effects to alleviate the lung injury induced by ventilation should be answered.

PPAR*γ*, a ligand-activated transcription factor, has effects on type 2 diabetes, atherosclerosis, and cancer [20]. Activation of PPAR *γ* by its ligands also reduces the expression of proinflammatory cytokines, such as tumor necrosis factor (TNF)-*α* and interleukin (IL)-1*β*, and its induction is mediated via NF-*κ*B and mitogen-activated protein kinase [21]. Some studies suggested that lung inflammation induced by LPS might be attenuated by RvD1, and the protective mechanisms might be ascribed to the selective reaction in the NF-*κ*B pathway [22, 23]. Nonetheless, whether RvD1 has a protective effect on VILI and what role PPAR*γ* plays in the inflammatory control of RvD1 have been poorly understood. In this study, we aimed to know if RvD1 exerts its protective effects in VILI by inhibiting NF-*κ*B via activating PPAR*γ*.

## 2. Materials and Methods

### 2.1. Animals

6-8-week-old male C57BL/6 mice (Experimental Animal Center of Wuhan University, China) weighing 20-25 g were kept in the specific pathogen-free environment (12/12 h of the light/dark cycle, 22-24°C), with free access to food and water. Animals were fasted 12 h before experiments but allowed water ad libitum. All animal experiments were approved by the Animal Care and Use Committee of Tongji Medical College of Huazhong University of Science and Technology, Wuhan, China.

### 2.2. Experimental Design

Mice were weighed and then anesthetized with ketamine(120 mg/kg, intraperitoneally) and xylazine (8 mg/kg, intraperitoneally). One-fourth of the initial dose of anesthetic drugs was supplied about every 1h to maintain anesthesia during the experimental period. The mice were fixed in a supine position on a heating blanket and were subjected to tracheotomy. A 20G vein catheter was inserted for mechanical ventilation (Model 863 Ventilator; Harvard, USA). Mice in all groups, except the sham group, had 40 ml/kg ventilation and 80 breaths/min for 4 hours as described previously [24] and underwent mechanical ventilation for 0 positive end-expiratory pressure.

**Figure 1:**
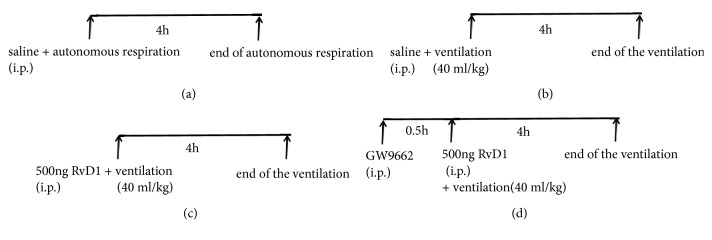
Mice were randomly divided into 4 groups. Sham group (a): mice was retained autonomous respiration and normal saline was injected intraperitoneally at the beginning of the experiment. High tidal volume group (b): normal saline was injected intraperitoneally at the beginning of the ventilation. 500ng RvD1 plus HV_T_ group (c): 500ng RvD1 was injected intraperitoneally at the beginning of the ventilation. PPAR-*γ* inhibitor group (d): GW9662 was given intraperitoneally 30 min before the start of mechanical ventilation and 500ng RvD1 was injected intraperitoneally at the beginning of the ventilation.

As shown in Figure 1, mice were randomly divided into 4 groups (n=7 per group): sham group (sham); high tidal volume group (HV_T_); 500ng RvD1 plus HV_T_ group (RvD1); PPAR-*γ* inhibitor group (GW9662).

RvD1 (Cayman Chemical Company, USA) was diluted with saline as per instructions and then injected intraperitoneally at the beginning of ventilation. The dosage of 500 ng/mouse was selected based on our own preexperimental data and previous studies [25]. Mice subjected to sham and HV_T_ group were injected with an identical volume of saline at the same time point. GW9662 (Santa Cruz, USA) was dissolved in 10% DMSO (no effect on mice) according to the instructions and administered a dose of 1 mg/kg intraperitoneally 30min before the start of mechanical ventilation. Both the time and the dose of administration are based on previous descriptions [26]. All experiments were carried out at least three times with different mice.

### 2.3. Measurement of PaO_*2*_

At the end of the experiment, the arterial blood (heparinized) from the abdominal aorta was analyzed for blood gas, and a blood gas analyzer was employed to measure the level of PaO_2_ in the blood.

### 2.4. Histological Analysis

The right lung tissue was fixed in 4% paraformaldehyde for 24 h and embedded with paraffin. Sections of 4 *μ*m thickness were made under an ordinary optical microscope and were HE-stained. The lung injury was rated on a scoring system developed by American Thoracic Society [27].

### 2.5. Lung Wet-to-Dry Weight Ratio

The right middle lung was weighed after the surface was cleaned with absorbent paper and then placed in an oven until the weight was constant. The W/D ratio was calculated as a measure of the severity of pulmonary edema.

### 2.6. Bronchoalveolar Lavage Analysis

Bronchoalveolar lavage was performed using three aliquots of 0.5mL phosphate-buffered saline (PBS) as previously described [19]. After being centrifugated at 12000 rpm at 4°C for 15min, the supernatant was collected and used for total protein concentration by BCA assay kit (Thermo Scientific). The sediment was resuspended in PBS, and the total number of BALF cells was determined by a hematocytometer. Cytospin (Thermo Shandon, Pittsburgh, Pennsylvania) was used to coat another part of the suspension at 800rpm for 10 minutes on a microscope slide. Wright Giemsa was used to stain the sections and count more than 300 cells under an optical microscope for histopathological examination and for differentiation of neutrophils and macrophages.

### 2.7. Cytokine Analysis in BALF

The concentrations of interleukin (IL)-1*β*, tumor necrosis factor (TNF)-*α*, IL-6, receptor for advanced glycation end-products (RAGE) were determined by enzyme-linked immunosorbent assays (IL-1*β*, TNF-*α*, IL-6 from Neobioscience, Shenzhen, China; RAGE from Ray Bio, USA )

### 2.8. Protein Extraction and Western Blot

The total protein of lung tissue was isolated with a protein extraction kit (Nanjing KeyGen Biotech Co., Ltd.) according to the manufacturer's protocol. Nuclear and cytoplasmic fractionations were performed using ER Nuclear and Cytoplasmic Extraction Reagents (Thermo Scientific, USA). Protein concentrations were measured with the BCA Protein Assay kit. The samples were subjected to SDS-polyacrylamide gel electrophoresis (PAGE) in 10% polyacrylamide gels. Immunoblotting was performed with antibodies to NF-*κ*B p65 or I*κ*B or PP AR-*γ* (Santa Cruz Biotechnology) then using HRP-labeled-goat anti-rabbit antibody. Image Lab was used for quantification.

### 2.9. Electrophoretic Mobility Shift Assay

The preparation of nuclear extract was carried out as previously described. The oligonucleotide probe corresponding to the consistent sequence of the binding site of ‘5-AGTTGAGGGGACTCCCAGGC-3' was synthesized and labeled with biotin. According to the instructions of commercial kits, electrophoretic mobility shift analysis (EMSA) was performed using the LightShift Chemiluminescent EMSA kit (Thermo Scientific, USA).

### 2.10. Statistical Analysis

All results are expressed as means ± standard errors of the means (SEM) and analyzed using GraphPad Prism (version 6.0, USA). One way ANOVA followed by the least significant difference post hoc test was used to assess differences between groups. A P < 0.05 was considered significant.

## 3. Results

### 3.1. RvD1 Attenuated Ventilator-Induced Lung Injury

**Figure 2:**
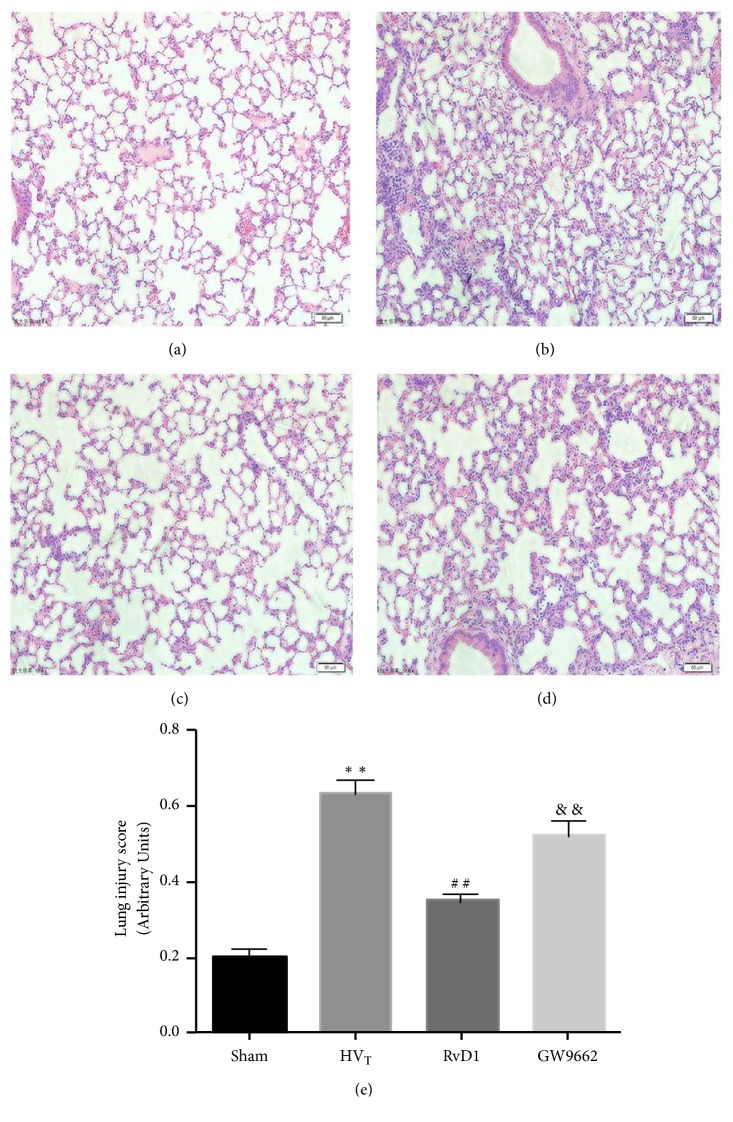
RvD1 attenuated lung injury scores in VILI mice. Representative hematoxylin-eosin staining pictures of lung tissue (magnification 200×). Sham group (a), HV_T_ group (b), RvD1 plus HV_T_ group (c), GW9662 plus RvD1 plus HV_T_ group (d), lung injury score (e). Data are presented as means ± SEM, n = 7. *∗∗* means P < 0.01 versus sham group; ## P < 0.01 versus HV_T_ group; && P < 0.01 versus the RvD1 group.

As shown in Figure 1(a), no significant change was observed in the tissue of the sham-operation group. Conversely, samples from HV_T_ animals were conspicuously damaged, suggesting a significant deterioration in inflammatory cell infiltration, alveolar interstitial edema, airspace, and transparent membranes being filled with protein fragments (Figure 1(b)). The pathological changes of the lung caused by HV_T_ ventilation were not obvious after treatment with RvD1 (Figure 1(c)). However, animals treated with GW9662 exhibited changes similar to those found in the HV_T_ animals (Figure 2(d)) and their lung injury scores were consistent with the pathohistological changes (Figure 2(e)).

### 3.2. RvD1 Improved Pulmonary Functions in Ventilator-Induced Mice

**Figure 3:**
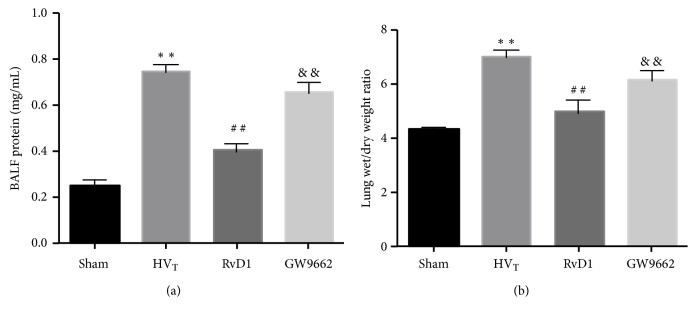
RvD1 ameliorated lung function in VILI mice. BALF protein content (a) and lung wet-to-dry ratio (b). Data are presented as means ± SEM, n = 7. *∗∗* means P < 0.01 versus sham group; ## P < 0.01 versus HV_T_ group; && P < 0.01 versus RvD1 group.

Lung injury was assessed by such measures as the ratio of lung wet/dry weight and protein levels in BALF and PaO_2_. Protein levels were used to reflect the microvascular permeability; the degree of pulmonary edema was measured by the lung wet/dry weight ratio; lung oxygenation capacity was evaluated in terms of the level of PaO_2_. Figures 3(a) and 3(b) show that there was no injury in the sham-operation group, whereas HV_T_ caused severe exudation and edema. The protein level in BALF and the W/D ratio were lowered by RvD1, but this action was partially abolished upon administration of GW9662.

**Figure 4:**
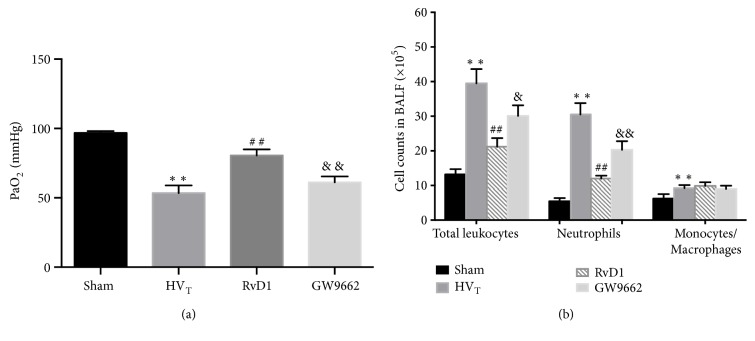
RvD1 improves oxygenation capacity of lung and decreased leukocyte recruitment and neutrophil infiltration in VILI mice; arterial oxygen pressures (a), cell counts in BALF (b). Data are presented as means ± SEM, n = 7. *∗∗* means P < 0.01 versus sham group; ## P < 0.01 versus HV_T_ group; && P < 0.01 versus RvD1 group.

Figure 4(a) shows that HV_T_, in general, caused significant changes in arterial oxygen partial pressure (PaO_2_). PaO_2_ was significantly lowered in HV_T_ group, whereas in the sham-operation group, PaO_2_ was normal. RvD1, in particular, substantially improved the oxygenation capacity. However, after administration of GW9662, the protective effect was diminished compared with the RvD1 group.

### 3.3. RvD1 Reduced Ventilator-Induced Neutrophil Infiltration

Figure 4(b) exhibits that the cell counts in BALF were constantly higher in the HV_T_ group than in the sham-operation group. Changes in RvD1-treated mice were different from those in the HV_T_ group, including total lymphocytes and neutrophils. RvD1-treated group reduced the recruitment of inflammatory cells to the lungs. On the contrary, there was no significant difference in the number of macrophages between RvD1-treated group and HV_T_ group. Our findings suggested that the increased white blood cells were principally neutrophils, and the RvD1, as a protector, worked also mainly on neutrophils while GW9662 could abolish this effect to some degree.

### 3.4. RvD1 Attenuated Inflammatory Response in Ventilator-Induced Mice

**Figure 5:**
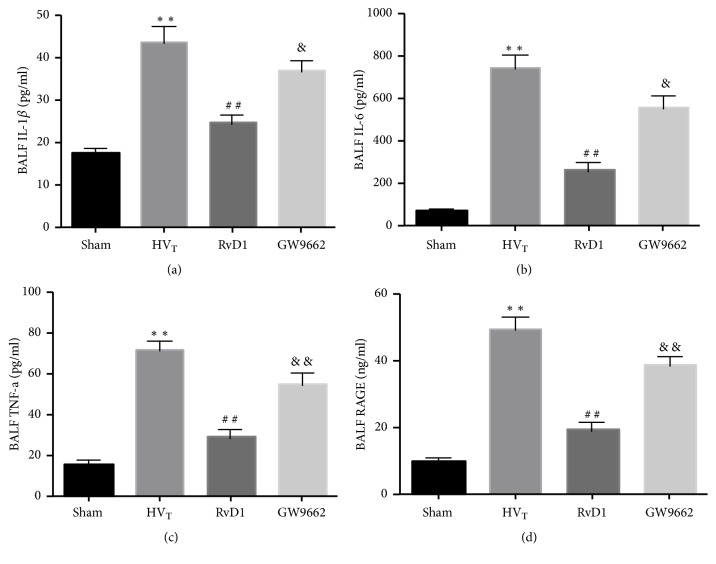
RvD1 reduced the production of inflammatory cytokines. IL-1*β* (a), IL-6 (b), TNF-*α* (c), RAGE (d). Data are presented as means ± SEM, n = 7. *∗∗* means P < 0.01 versus sham group; ## P < 0.01 versus HV_T_ group; && P < 0.01 versus RvD1 group.

Compared to the sham-operation group, HV_T_ significantly increased the production of cytokines. RvD1 markedly downregulated the expression of IL-1*β* (Figure 5(a)), IL-6 (Figure 5(b)), TNF-*α* (Figure 5(c)), and RAGE (Figure 5(d)). In addition, the concentration of inflammatory cytokines was dramatically increased upon treatment with GW9662.

### 3.5. Effect of RvD1 on PPAR*γ*, I*κ*B*α*, and NF-*κ*B p-65 Subunit Expression in Ventilator-Induced Lung Injury

**Figure 6:**
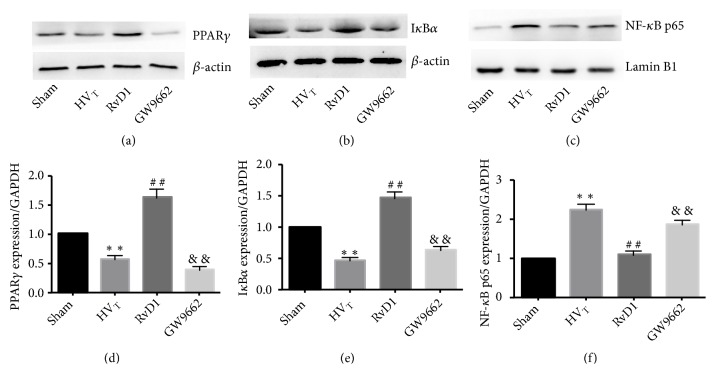
RvD1 affects the expression of I*κ*B*α*, NF-*κ*B p65 subunit, and PPAR*γ* in VILI mice. Western blot analysis for PPAR*γ* (a), I*κ*B*α* (b), and NF-*κ*B p65 (c). PPAR*γ* protein levels normalized by GADPH (d), I*κ*B*α* protein levels normalized by GADPH (e), NF-*κ*B p65 protein levels normalized by GADPH (f). Data are presented as means ± SEM, n = 7. *∗∗* means P < 0.01 versus sham group; ## P < 0.01 versus HV_T_ group; && P < 0.01 versus RvD1 group.

As shown in (Figures 6(a) and 6(b)), HV T ventilation upregulated the expression of PPAR*γ* and NF-*κ*B p65 subunit. Treatment with RvD1 suppressed ventilation-activated signaling molecules, and the effect was reversed by GW9662. The levels of I*κ*B*α* were downregulated in response to HV_T_ ventilation compared with the sham-operation group. However, RvD1 treatment inhibited ventilation-induced I*κ*B*α* degradation, which was partially blocked after GW9662 was given.

### 3.6. RvD1 Reduced DNA-Binding Activity of NF-*κ*B in Ventilator-Induced Mice

**Figure 7:**
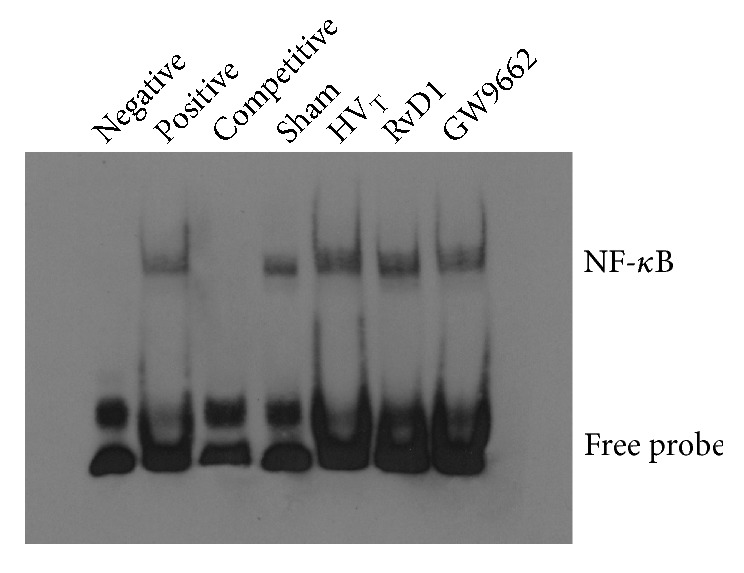
RvD1 decreased DNA-binding ability of NF-*κ*B in VILI mice. EMSAs were performed to detect the DNA-binding activity of NF-*κ*B in nuclear extracts. Data are presented as means ± SEM, n = 7. *∗∗* means P < 0.01 versus sham group; ## P < 0.01 versus HV_T_ group; && P < 0.01 versus RvD1 group.

Deoxyribonuclease-binding activities of NF-*κ*B were markedly increased in the HV_T_ animals (Figure 7). This change was obviously relieved in the RvD1-treated animals, whereas the NF-*κ*B DNA-binding activity was significantly enhanced upon administration of GW9662.

## 4. Discussion

Mechanical ventilation (MV) is considered an indispensable supportive treatment for respiratory failure. However, a major concern with mechanical ventilation is VLLI, which warrants effective addressing. In this study, the VILI model was successfully established, according to a previously described technique [24]. Our model developed a disrupted alveolar-capillary barrier, which mimicked the pathological changes of VILI (the interstitial edema, increased microvascular permeability, the release of proinflammatory cytokines, and neutrophil infiltration) and had all the signs indicative of its success.

By employing the model, we demonstrated that RvD1 exerted a strong protective effect on acute lung inflammation. Our experimental results revealed that the use of RvD1 could significantly relieve the pathological changes of lungs, pulmonary edema, leukocyte infiltration, and release of proinflammatory mediators. The main pathological changes of lung inflammation involve the disruption of epithelial barrier [28]. Serum breaks through the impaired epithelial cells and infiltrates into lung tissues, resulting in increased protein level in BALF and development of pulmonary edema. Protein in BALF can serve as a measure for pulmonary vascular permeability and is an indicator of acute lung injury [29]. In this study, RvD1 could reduce protein concentration in the BALF, suggesting that RvD1 can effectively protect pulmonary vascular endothelial and thereby maintain the vascular permeability in VILI-induced inflammation. Since edema reflects the severity of inflammation [30], we used W/D ratios as a measure for the degree of edema. Our results showed that RvD1 could significantly inhibit VILI-induced pulmonary edema, suggesting that RvD1 possesses a powerful antiedematogenic effect.

High tidal volume ventilation has been found to elevate the plasma cytokine level [31, 32], and the increased proinflammatory cytokines, such as factor TNF-*α*, and interleukins (IL-1*β*, IL-6) [27, 28], believed to be the markers of inflammation, are associated with an unfavorable prognosis in patients with VLII [33]. Our results exhibited that RvD1 evidently decreased the levels of IL-1*β*, TNF-*α*, IL-6 in BALF of HV_T_ animals and the findings were coincident with previous results [34]. RAGE is highly expressed in the lungs, primarily on the basolateral membrane of alveolar type I cells [31], and several studies demonstrated that RAGE could be used as a biomarker for lung injury both in animal models and in clinical practice [32, 35]. Further study showed that RAGE signaling was conducive to the proinflammatory status or process in ventilator-induced lung injury [36]. Our study showed that treatment with RvD1 lowered the expression of RAGE and that RvD1 could reduce proinflammatory factors of the early phase of inflammation, especially in VILI.

The neutrophil infiltration plays an essential role in proinflammatory processes in VILI [37]. The main histological feature in resolution is the loss of neutrophils from the local inflamed sites. [38]. Our results showed that after treatment with RvD1, fewer neutrophils infiltrated into the alveolar space, as evidenced by histological analysis of lung tissue and the cell counts in BALF. RvD1 may restrict total leukocytic infiltration through the same mechanisms by which RvD1 not only reduces inflammation but also promotes resolution back to noninflammatory state [38]. At the tissue level, several mechanisms might be at work.

(1) It might reduce the levels of proinflammatory mediators, just like prostaglandins [39]; (2) it might prevent neutrophils from entering the inflamed area; (3) the production of nitric oxide stimulated by endothelial cells might block the adhesion of leukocytes to vascular endothelial cells and stimulate macrophages to phagocytize apoptotic neutrophils [38]; the orphan receptor G-protein-coupled receptor 32 (GPR32) and the lipoxin A4/Annexin-A1 receptor formyl-peptide receptor 2 (FPR2/ALX) were reported as mediators in the process of RvD1 regulated human neutrophil recruitment [39]. GPR32 and FPR2/ALX are expressed on human cells which are directly related to inflammation, including neutrophils, lymphocytes, macrophages, and monocytes [40]. It is generally believed that RvD1 binds to GPR32 during periods of homeostasis, while RvD1 interacts with ALX/FPR2 during periods of resolving inflammation. Recently, studies show that RvD1 could promote the transformation of macrophage phenotype from proinflammatory to a proresolving M2-like phenotype by triggering both GPR32 and FPR2/ALX [41]. GPR32 and ALX/FPR2 receptors engaged RvD1 to inhibit the secretion of cytokines by CD8^+^T cells, and CD4^+^T cells indicate that RvD1 exerts its anti-inflammatory effects through binding to these two receptors. As a promiscuous G-protein coupled receptor (GPCR), FPR2/ALX participates in promoting the resolution of inflammation, such as neutrophil extravasation limitation, neutrophil apoptosis induction, and apoptotic cell phagocytosis promotion acts in the monocyte recruitment modulation.

PPAR*γ* is involved in the lipid metabolism, cell differentiation, and, more importantly, inflammatory processes, including cytokine production and antigen presentation [42]. Previous studies have suggested that the PPAR*γ* pathway plays a vital role in developing multiform of damage shown in VILI [43]. After toll-like receptors (TLRs) recognize the antigen, they activate the NF-*κ*B pathway to regulate the expression of cytokines and other proteins with antimicrobial activity or signaling properties [44]. Multiple steps in the NF-*κ*B signaling pathway could be inhibited by PPAR *γ* ligands, and once activated, they lead to the inhibition of NF- *κ*B [45]. GW9662, a potent irreversible and selective antagonist of PPAR*γ*, is known to be a useful tool for studying the role of PPAR*γ* in biological processes [46]. In our study, we found that treatment with GW9662 could partially reduce the inhibition of NF-*κ*B activation induced by RvD1, indicating that RvD1 attenuated VILI possibly through PPAR*γ*/NF-*κ*B pathway. All of these findings indicated that the inflammation resolution induced by RvD1 might depend on the NF-*κ*B signaling pathways.

## 5. Conclusions

Our results indicated that RvD1 could limit lung inflammation in VILI mice and suppress the NF-*κ*B p-65 subunit. We also demonstrated that the inhibition of NF- *κ*B activation might, to some degree, be partially reduced through PPAR*γ*. On the basis of the results, we are led to conclude that RvD1 can reduce the inflammatory response in VILI by activating PPAR-*γ* and inhibiting NF-*κ*B signaling pathways.

## Data Availability

The data used to support the findings of this study are available from the corresponding author upon request.
